# Unravelling the complexities of DNA-PK activation by structure-based mutagenesis

**DOI:** 10.21203/rs.3.rs-3627471/v1

**Published:** 2023-12-13

**Authors:** Christopher J. Buehl, Noah J. Goff, Mariia Mikhova, Steven W. Hardwick, Thomas L. Blundell, Mauro Modesti, Jens C. Schmidt, Amanda Chaplin, Katheryn Meek

**Affiliations:** 1 College of Veterinary Medicine, Department of Microbiology & Molecular Genetics, Department of Pathobiology & Diagnostic Investigation, Michigan State University, East Lansing, MI 48824, USA.; 2 Department of Biochemistry and Molecular Biology, Michigan State University, East Lansing, MI, U.S.A; 3 Institute for Quantitative Health Sciences and Engineering, Michigan State University, East Lansing, MI, U.S.A; 4 CryoEM Facility, Department of Biochemistry, University of Cambridge, Sanger Building, Tennis Court Road, Cambridge CB2 1GA, United Kingdom.; 5 Department of Biochemistry, University of Cambridge, Sanger Building, Tennis Court Road, Cambridge, CB2 1GA, United Kingdom.; 6 Cancer Research Center of Marseille, Department of Genome Integrity, CNRS UMR7258, Inserm U1068, Institut Paoli-Calmettes, Aix Marseille Univ, Marseille, France; 7 Department of Obstetrics, Gynecology, and Reproductive Biology, Michigan State University, East Lansing MI, U.S.A; 8 Leicester Institute for Structural and Chemical Biology, Department of Molecular and Cell Biology, University of Leicester, Leicester, UK.

## Abstract

It has been known for decades that the DNA-dependent protein kinase (DNA-PK) is only an active serine/threonine protein kinase when it is bound to a DNA double-stranded end; still, the molecular details of how this activation is achieved have remained elusive. The recent surge in structural information for DNA-PK complexes has provided valuable insights into the process of DNA end recognition by DNA-PK. A particularly intriguing feature of this kinase is a region of the protein that can transition from a seemingly structurally disordered state to a single alpha-helix that traverses down the DNA binding cradle. The DNA-PK bound DNA end of the DNA substrate engages with and appears to split around this helix which has been named the **D**NA **E**nd **B**locking helix (DEB). Here a mutational approach is utilized to clarify the role of the DEB, and how DNA ends activate the enzyme. Our data suggest two distinct methods of kinase activation that is dependent on the DNA end chemistry. If the DNA end can split around the helix and stabilize the interaction between the DNA end and the DEB with a recently defined **H**elix-**H**airpin-**H**elix (HHH) motif, the kinase forms an end-protection monomer that is active towards DNA-PK’s many substrates. But if the DNA end cannot stably interact with the DEB [because of the DNA end structure, for instance hairpins, or because the DEB has been disrupted by mutation], the kinase is only partially activated, resulting in specific autophosphorylations of the DNA-PK monomer that allows nucleolytic end-processing. We posit that mutants that disrupt the capacity to stably generate the DEB/HHH DNA end-interaction are inefficient in generating the dimer complex that is requisite for NHEJ. In support of this idea, mutations that promote formation of this dimer partially rescue the severe cellular phenotypes associated with mutation of the DEB helix.

## Introduction

DNA, the blueprint of life, is surprisingly easy to damage. Unlike single-stranded DNA breaks (SSB) that are relatively easily resolved because the DNA is held together by the second strand, DNA double strand breaks (DSBs) often have a lethal impact. Two major DNA double-strand break repair (DSBR) pathways [homologous recombination (HR) and non-homologous end joining (NHEJ)] have evolved and exist in all higher vertebrates^1^. NHEJ is the predominant pathway in higher vertebrates and is active throughout the cell cycle, whereas HR which is dependent on a sister chromatid for homology directed repair is limited to the S and G2 phases of the cell cycle^2,3^.

**Fig.1.**
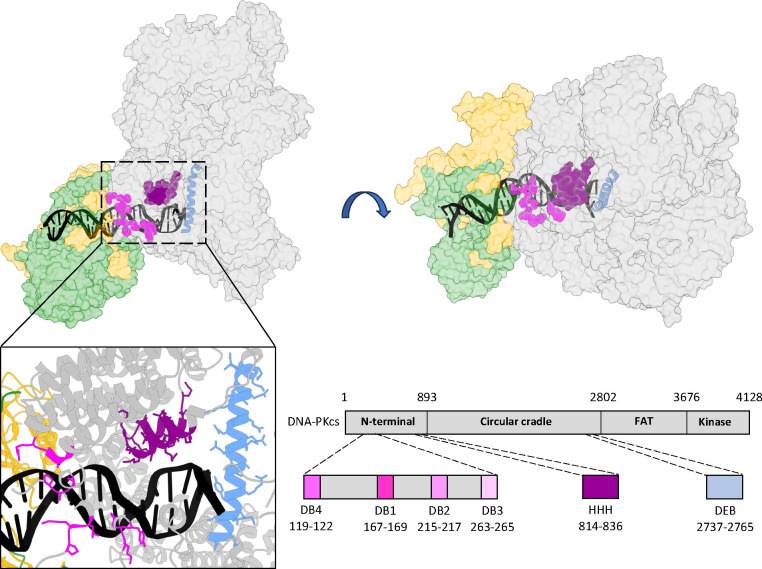
DNA/protein interaction sites that impact DNA-PK enzymatic activation. Ribbon diagram of DNA bound DNA-PK monomer (PDB 7Z87). DNA-PKcs is shaded grey, Ku70 yellow, Ku80 green. Lower right, cartoon depicting residues in DNA-PKcs that interact with DNA and impact kinase activation. Color coding of these sites in cartoon corresponds to that in ribbon diagram.

NHEJ is initiated when the Ku70/80 heterodimer engages a DSB and associates with the large catalytic subunit of the DNA-dependent protein kinase (DNA-PKcs)^4–7^. Although it has been known for decades that the DNA dependent protein kinase is fully activated when DNA-PKcs associates with a DNA double-stranded end bound to Ku^6–8^, the molecular events that promote activation have not been defined. Recently, there has been an explosion of new structural and functional information (from cryo-EM and mutational studies) of NHEJ complexes that provide snapshots of distinct steps of the joining mechanism^9–18^. These studies have highlighted several DNA-protein interactions that could potentially impact kinase activation (Fig. 1). Four distinct patches in the N-terminal region of DNA-PKcs interact directly with substrate DNA (DNA binding 1–4; DB1, DB2, DB3, DB4; highlighted pink, Fig. 1); these interactions are not restricted to the DNA ends, but extend 15 base pair (bp) internal to the DNA end that, along with Ku’s DNA interaction, create a DNA binding “cradle” that encompasses 29bp (consistent with previous biochemical studies)^19,20^ (Fig. 1). A novel DNA-protein interface, the **D**NA **E**nd-**B**locking (DEB) helix (highlighted blue, Fig. 1), has been proposed to protect DNA ends^12^. The DEB helix (an unpacked helix) traverses through the DNA binding cradle and is only observed in a subset of DNA-bound DNA-PK complexes; in these structures, the DEB is generated from a structural transition of a region in DNA-PKcs that is disordered in previous structures. Interestingly, the DNA 5’ and 3’ termini appear to split around the DEB. Finally, a new motif designated **H**elix-**H**airpin-**H**elix (HHH) motif (highlighted purple, Fig. 1) is proposed to allosterically signal kinase activation. The focus of this study is to define the function(s) of complexes that initially recognize the DNA double-stranded ends and promote kinase activity. We further investigate how differences in end detection impact how DNA-PK promotes either end protection or nucleolytic processing.

## Results

### Four patches in DNA-PKcs within the N-HEAT region function redundantly to form the DNA cradle in the DNA-PK holoenzyme; this DNA binding cradle is essential for kinase activation.

**Fig. 2.**
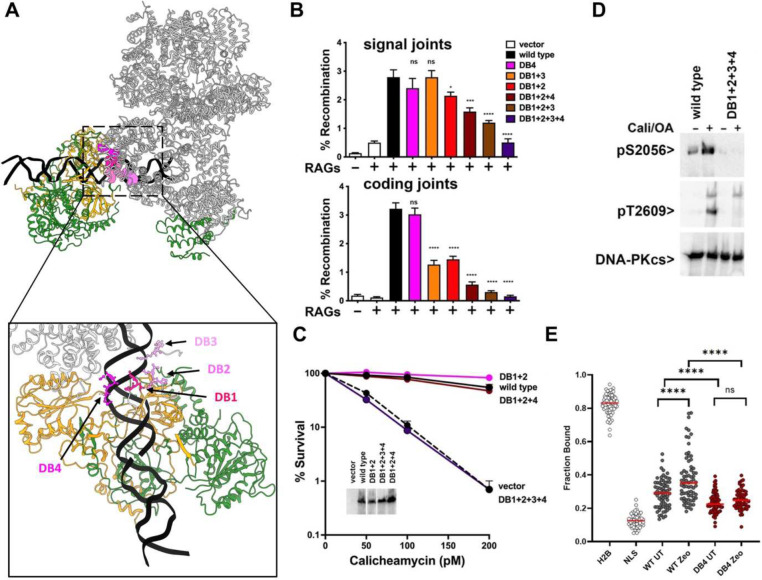
Four patches in DNA-PKcs within the N-HEAT region function redundantly to form the DNA cradle in the DNA-PK holo-enzyme; this DNA binding cradle is essential for kinase activation. (A) Ribbon diagram depicting position of four DNA cradle protein/DNA interaction sites (PDB 7K1N). (B)The fluorescent substrate 290-Crimson/ZS and 289-Crimson/ZS were utilized to detect coding and signal end joining of RAG-induced DSBs. Percent recombination of episomal fluorescent coding-end joining substrate in V3 cells transiently transfected with wild-type or mutant DNA-PKcs expression constructs as indicated. Error bars indicate SEM comparing wild type to each mutant from six independent experiments. ****, P<.0001; ***, P<.001. (C) V3 clonal transfectants expressing wild-type or mutant DNA-PKcs as indicated were plated at cloning densities into complete medium with increasing doses of calicheamicin. Colonies were stained after eight days, and percent survival was calculated. Error bars represent the standard error of the means for six independent experiments. (D) Immunoblot analyses using indicated antibodies of cell extracts from indicated V3 transfectants treated or not with 40nM calicheamicin and 1uM Okadaic acid for thirty minutes. (E) Plot of the Fraction Bound of DNA-PKcs variants in live cell single-molecule imaging experiments under untreated conditions and post-zeocin treatment that were analyzed using a two-state model of diffusion. Each data point represents the fraction bound of each protein in an individual cell (N = 3 biological replicates, n = 20 cells per replicate and condition, Red bar = median). Data were analyzed by two-way ANOVA with Tukey’s posthoc test (**** = p < 0.0001).

In monomeric DNA-PK structures, many DNA/protein interactions are apparent. Because it seems likely that DNA-PK is initially activated as a monomer, we first interrogated the functional role of these DNA-protein interactions. There are four distinct patches within the N-terminal HEAT region (N-HEAT) that bind the minor groove of the 5th to 15th bp of DNA^9^ just internal to the DNA terminus consistent with previous studies^19^. These patches (depicted in different shades of pink, Fig. 2A) include residues 167–169/PDT (DNA-binding 1, DB1), residues 215–218/PKLP (DB2), residues 263–265/KRY (DB3), and residues 119–122/RAAK (DB4). Ku does not contact the DNA end, but stabilizes the DNA-PKcs:DNA interaction and extends the DNA binding cradle another 14bp. DNA-PKcs expression constructs that disrupt each patch alone or in various combinations (see Sup. Table 1) were used in episomal VDJ coding and signal joining assays using the V3 DNA-PKcs deficient CHO cell strain (Fig. 2B). Mutation of one or two of the four DNA cradle interfaces in DNA-PKcs has little or modest impact on VDJ signal or coding end joining. However, mutations that disrupt three patches have a more pronounced impact on joining, and ablation of all four abrogates coding end joining, and severely impairs signal end joining. Stable transfectants were established that express a subset of these mutants; cell strains expressing DNA-PKcs with two, or three of the DNA cradle patch mutants were similarly resistant to calicheamicin as compared to cells expressing wild-type DNA-PKcs (Fig. 2C). [Here and in all experiments, at least two independent clones were tested for each mutant, but for clarity only a single clone is presented.] In contrast, cells expressing DNA-PKcs in which all four of the DNA cradle interfaces were mutated were as calicheamicin-sensitive as cells completely lacking DNA-PKcs.

DNA-PK’s most relevant target is itself; autophosphorylation at two major clusters (termed ABCDE and PQR) reciprocally regulates end-processing^21–23^. To gauge DNA-PK enzymatic activity in living cells, DNA-PKcs autophosphorylation was assessed. Cells expressing wild-type DNA-PKcs or the quadruple mutant were treated for 30 minutes with 40nM calicheamicin to induce DSBs and 1ug/ml okadaic acid to inhibit protein phosphatases; cell extracts were isolated and analyzed by immunoblot to detect DNA-PKcs autophosphorylations. As can be seen (Fig. 2D), whereas T2609 (an ABCDE site) and S2056 (a PQR site) autophosphorylations are robustly induced in wild-type cells, these autophosphorylations are absent in cells expressing the DNA cradle quadruple mutant. To analyze the recruitment of DNA-PKcs to DNA breaks in living cells, we employed our recently developed single-molecule imaging assay of 3xFLAG-HaloTagged DNA-PKcs^24^. In these experiments the fraction of static DNA-PKcs molecules (Fraction Bound) is used as a readout of the binding of DNA-PKcs to zeocin induced DSBs. As controls, we used the HaloTag protein fused to a nuclear localization signal (Halo-NLS) to represent a freely diffusing molecule in the nucleus, whereas HaloTagged histone H2B (Halo-H2B) was used as constitutively chromatin bound control protein (Fig. 2E). As expected, zeocin exposure induces chromatin association of wild type DNA-PKcs; in contrast, the DNA cradle mutant fails to associate with chromatin in response to zeocin-induced DSBs (Fig. 2E). In addition, the basal level of chromatin recruitment is reduced by mutation of the DNA cradle (Fig. 2E). We conclude that mutants disrupting the extensive interaction of DNA-PKcs with DNA in the DNA binding cradle abrogate DNA-PK’s capacity to bind DSBs in living cells, abrogating kinase activity. These data establish that stabilization of the DNA end by the extensive DNA binding cradle (and not just DNA-end detection) is necessary to initiate kinase activation.

In structures where the DEB is disordered, in addition to the four DNA binding cradle patches, the DNA end clearly interacts with specific regions in the N-HEAT region, with the 5’ end interacting with residues 356–357/NK and D405 and the DNA 3’-OH being covered by residues 518–520/KWK^9^. [It should be noted that lack of a structured DEB cannot be attributed to low structural resolution; the DEB is not structured in a number of high-resolution DNA-PK structures^9^.] It is unclear what signals the structural transition that gives rise to the DEB helix, and we considered that these DNA end interactions might promote formation of the DEB and impact function. DNA-PKcs expression vectors that disrupt each of these interaction sites alone, as well in various combinations (see Sup. Table 1) were generated and tested in episomal VDJ assays. As can be seen (Sup. Fig. 1), ablation of any of these interaction sites has no significant impact on joining of RAG-induced DSBs on episomal substrates. Numerous combinations and variations of these mutations were constructed and tested, but none had a significant impact on end-joining using episomal assays. These data suggest that these DNA end interactions do not impact DNA-PK activation or function, and it remains unclear what promotes formation of the ordered DEB helix.

### DEB mutant DNA-PK “senses” all DNA ends like hairpin ends.

The enormous 4128 residue DNA-PKcs polypeptide is composed largely of well-ordered HEAT repeats with the relatively small lipid-like kinase domain positioned at the C-terminus of the polypeptide. Previously, a psi-pred analysis of DNA-PKcs predicted that ~200 residues that includes the functionally critical “ABCDE” autophosphorylation sites^23^ was likely disordered; this is the same region that includes the DEB helix that can be visualized in both monomeric and dimeric structures^9–12,15–18,25^. Exactly what promotes the initial transition from a disordered region to the lone alpha helix DEB is not clear. As noted above, the DEB helix traverses through the DNA cradle and the DNA 5’ and 3’ ends split around the helix. Chen et al. predicted that the DNA end would be sequestered from DNA modifying enzymes whilst engaged with the DEB^12^. Liu et al. demonstrated that in the presence of ATP, blunt DNA ends favor formation of DNA-PK complexes containing the DEB helix, whereas hairpin ends (that cannot split around the helix) exclusively promote formation of the ABCDE phosphorylated complex^10^. It is important to note that the DEB helix is disordered when ABCDE sites (2609–2647) are phosphorylated. Phosphorylated ABCDE sites are repositioned by binding basic residues in the M-HEAT region, disrupting the closely connected DEB helix (2737–2765) and opening of the DNA binding cradle to promote end-processing by Artemis^10,16^.

The splitting of the duplexed DNA ends around the helix suggests several potential roles: 1) protection of the end, 2) detection of the end to regulate whether nucleolytic or fill-in end-processing is used, 3) promoting kinase activation. To test whether DNA end-engagement by the DEB helix impacts how DNA-PK is activated, Flag-Halo-tagged wild type DNA-PKcs (which substantially complements the radiosensitivity of V3 cells, Sup. Fig.2A), YARK>AAAA, and ABCDE>Ala mutant proteins were isolated from stably transfected V3 cells (Sup Fig. 2B). Purified DNA-PKcs in combination with purified human Ku were utilized in *in vitro* kinase assays. As reported previously^10,26^, open DNA ends (Cla fragment, C) fully activate wild type DNA-PK to phosphorylate both ABCDE (T2609) and PQR (S2056) autophosphorylation sites, and other substrates (XRCC4); in contrast, hairpin DNA ends (TelN fragment, T) only promote autophosphorylation of ABCDE sites by wild type DNA-PK. This is consistent with cryo-EM studies showing exclusive formation of ABCDE phosphorylated/nuclease competent DNA-PK monomers by hairpin DNA ends^10^. The YARK>AAAA mutant responds to open or hairpin DNA ends identically, and indistinguishable from hairpin activation of wild type DNA-PK. These data show that if the DNA end cannot stably interact with the DEB, either because of the DNA end structure or because the DEB has been disrupted by mutation, the kinase is only partially activated, promoting only ABCDE autophosphorylation.

Tethering of phosphorylated ABCDE sites to the M-HEAT region results in kinase inactivation because of DNA-PKcs dissociation from Ku bound DNA^10^. The ABCDE>Ala mutant responds to open and hairpin DNA ends similarly to wild type DNA-PK; as with wild-type DNA-PKcs, hairpin ends are not efficient in induction of XRCC4 and 2056 phosphorylation, although the ABCDE>ala mutant is somewhat more proficient than wild type for XRCC4 phosphorylation. These data suggest that the inactivation of the kinase after detection of hairpin DNA ends does not rely solely on tethering of the phosphorylated ABCDE sites to the M-HEAT region, and we conclude that the inability of hairpin ends to stably interact with the DEB directly contributes to partial kinase activation in response to hairpin ends. In sum, these data suggest that the DEB helix is central to the activation of DNA-PK; surprisingly activation occurs in two ways. If DNA-PK interacts stably with a DNA end by interactions in the DNA binding cradle and 1) the DEB “detects” and interacts with an open DNA end, DNA-PK is fully activated, phosphorylating both ABCDE and PQR sites as well as its other many targets, and the complex protects the DNA end, or 2) the DEB does not “detect” and interact with the DNA end; in this case DNA-PK is partially activated to specifically autophosphorylate the ABCDE sites that results in end release (and accessibility to nucleases), ensuring kinase inactivation.

### End processing is dysregulated if the DEB helix is disrupted by mutation.

**Fig. 4.**
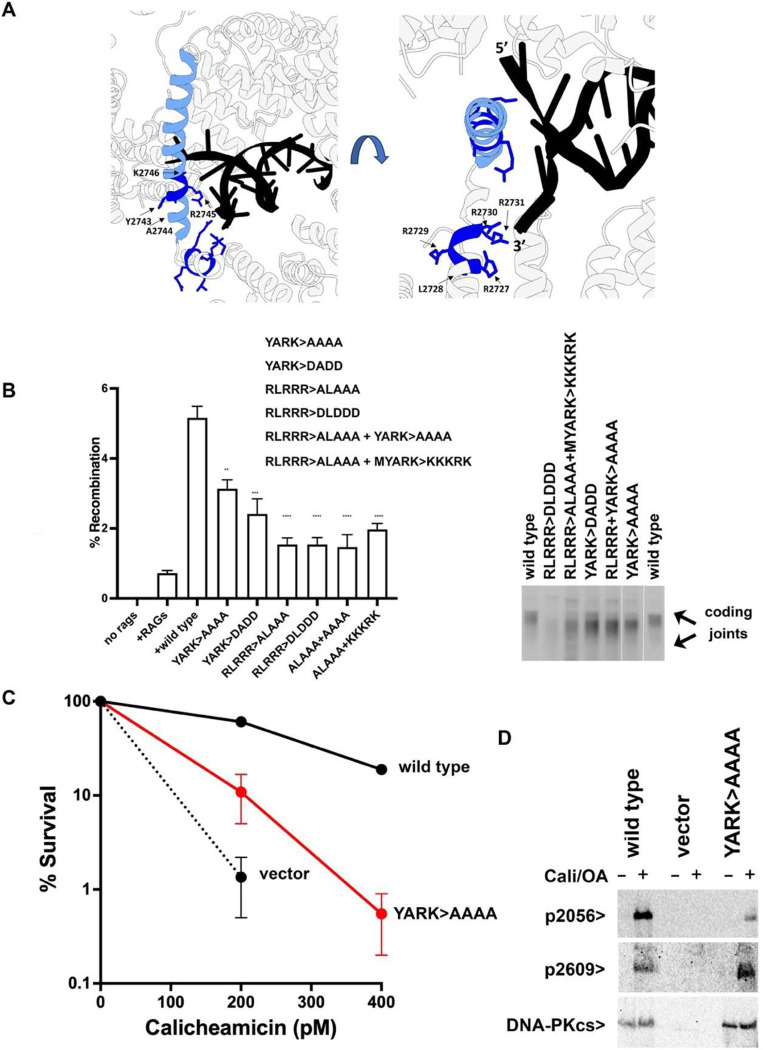
Mutation of the DEB helix promotes T2609 autophosphorylation and dysregulates nucleolytic end processing. (A) Ribbon diagram of DNA-PK (PDB 7NFC). DNA-PKcs is shown in grey; the DEB is shown in light blue, and the positively charged patch (2727–2731) is shown in dark blue. (B) (left) The fluorescent substrate 290-Crimson/ZS was utilized to detect coding end joining of RAG-induced DSBs as described in Fig. 2. Error bars indicate SEM comparing wild type to each mutant from three independent experiments. ****, P<.0001; ***, P<.001. (right) 2.5% agarose electrophoresis of PCR amplification of coding joints from AT coding joint substrate from V3 stable transfectants expressing wild-type or mutant DNA-PKcs and then transfected with substrate and RAG expression constructs. Cells were harvested 72 hours after transfection; coding joint substrate was isolated by alkaline lysates, followed by PCR for coding joints. (C) V3 clonal survival assays as in Fig. 2. (D) Immunoblot analyses using indicated antibodies of cell extracts from indicated V3 transfectants treated or not with 40nM calicheamicin and 1uM Okadaic acid for thirty minutes.

Since all DNA ends detected by the YARK>AAAA mutant would theoretically promote only ABCDE phosphorylation and end-processing DNA-PK complexes, it would seem likely that end-processing would be dysregulated in cells expressing this mutant, and we next focused on determining the impact of DEB mutation in living cells. From the cryo-EM structures, in addition to interaction of the DNA end with 2743–2746 (YARK, colored light blue, Fig. 4A), a positively charged patch 2727–2731 (RLRRR, colored in dark blue, Fig. 4A) interacts with the 3’ end, and we considered that this interaction might promote more stable association of the DNA end with the DEB. Five additional mutants were constructed including harsher mutations of 2743–2746 (YARK) alone or in combination with substitutions in residues 2727–2731 (RLRRR) (Sup. Table 1). All six mutants similarly impair VDJ coding joints in episomal assays (Fig. 4B). Coding joints were PCR amplified from these assays and analyzed by agarose gel electrophoresis (Fig. 4B, right panel). Coding joints isolated from cells expressing any of the YARK or RLRRR mutants are clearly shorter than coding joints amplified from cells expressing wild-type DNA-PKcs. PCR amplified coding joints from cells transfected with wild type DNA-PKcs or the YARK>AAAA mutant were purified and subjected to amplicon sequencing (Sup. Fig. 3). Here, 0 nucleotides lost is designated as having maintained the four base-pair hairpin overhangs (P segments) from either coding end. Joints isolated from cells expressing the YARK>AAAA mutant have more nucleotide loss as well as an increase in use of short sequence homologies at the junctions. We conclude that mutation of the DEB helix dysregulates end-processing.

Cell clones expressing the YARK>AAAA mutant were established (Fig. 4C). Cells expressing the YARK>AAAA are substantially more sensitive to calicheamicin than cells expressing wild-type DNA-PKcs but are slightly more resistant than cells expressing no DNA-PKcs. As in the *in vitro* kinase assays, the YARK>AAAA mutant autophosphorylates S2056 less efficiently than wild-type DNA-PKcs, but autophosphorylates T2609 more robustly than wild-type DNA-PKcs. The decrease in 2056 phosphorylation in living cells is less dramatic than in the kinase assays; this may be explained by the absence of XLF in the i*n vitro* assays. We and others have shown that XLF is essential for efficient 2056 phosphorylation, a PQR phosphorylation that occurs in trans, likely within the XLF-dependent dimer^17,27,28^. Increased phosphorylation of the ABCDE sites (like T2609) is consistent with increased nucleolytic activity of the YARK>AAAA mutant (Fig. 4C). We conclude that mutations in the DEB helix impair its ability to stably bind and split the bound DNA ends -- just like hairpin DNA ends; and like hairpin ends that specifically promote ABCDE phosphorylation and Artemis activation^10,26^, the DEB mutant also promotes ABCDE phosphorylation and nucleolytic DNA-PK complexes.

### The DEB helix and HHH motif cooperate to activate DNA-PK.

**Fig. 5.**
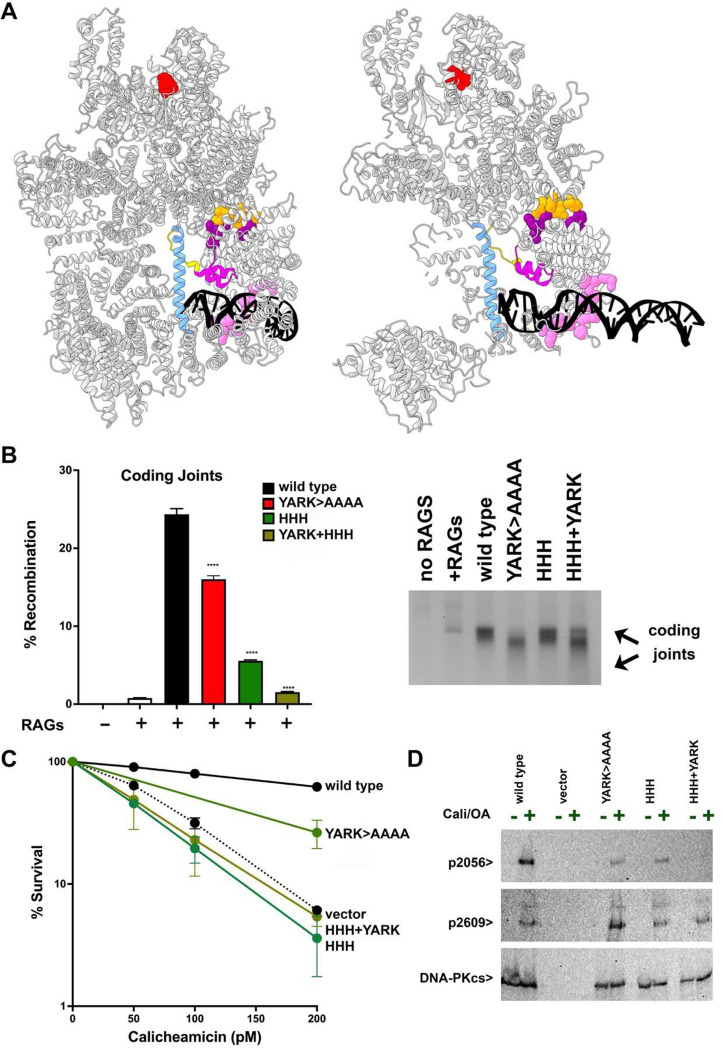
The DEB helix and HHH motif cooperate to activate DNA-PK. (A) Ribbon diagram of DNA-PK(PDB 7Z87). The DEB is colored light blue; HHH is colored magenta; DNA cradle patches are colored pink; N-terminal residues that interact with HHH are colored purple); flexible peptide (residues 837–746) is colored yellow; motif in FAT domain is colored orange; the catalytic site is colored red. (B) (left) The fluorescent substrate 290-Crimson/ZS was utilized to detect coding end joining of RAG-induced DSBs. Error bars indicate SEM comparing wild type to each mutant from four independent experiments. ****, P<.0001; ***, P<.001. (right) 2.5% agarose electrophoresis of PCR amplification of coding joints from AT coding joint substrate from V3 stable transfectants expressing wild-type or mutant DNA-PKcs. (C) V3 clonal survival assays. (D) Immunoblot analyses using indicated antibodies of cell extracts from indicated V3 transfectants treated or not with 40nM calicheamicin and 1uM Okadaic acid for thirty minutes.

Using a DNA-PK inhibitor, Liang and Blundell described DNA-PK monomer structures in both intermediate and full states of activation^15^, providing an elegant molecular explanation for the allosteric activation of DNA-PK. Their structures suggested that allosteric activation can be explained through conformational changes caused by interaction of a helix-hairpin-helix motif (HHH motif, residues 816–836, colored magenta, Fig. 5A) in the N-HEAT region, with the DNA (just across from binding by cluster 3 in the DNA binding cradle) and the DEB (colored light blue). The four patches in N-HEAT that form the DNA binding cradle are shaded light pink in Fig. 5A. When the HHH motif binds the DNA end (split around the DEB), an interaction on the opposite face of the HHH motif (colored orange) with the extreme N-terminus of DNA-PKcs is stabilized. This stabilization (which is likely communicated by the flexible peptide (residues 837–746, colored yellow) results in dramatic conformational changes of the FAT domain that is mediated by an interaction between the extreme N-terminus (colored purple) with the FAT domain (colored orange); this is proposed to promote kinase activation.

To test this proposed mechanism of kinase activation, a mutant designed to disrupt the interaction of the HHH motif with the DNA-phosphate backbone of the DNA was constructed. Four residues that directly interact with DNA were substituted with alanine [R820A, K824A, K832A, and H833A]; constructs encoding this mutant alone or in combination with the YARK>AAAA were prepared. Whereas the HHH mutant has a marked deficit in coding end joining, the YARK+HHH mutant is almost as deficient in joining as cells that lack DNA-PKcs (Fig. 5B). Coding joints were PCR amplified from these assays and analyzed by agarose gel electrophoresis (Fig. 5B). As before, coding joints amplified from cells expressing the YARK>AAAA mutant are markedly shorter than joints from wild type cells; although coding end joining is markedly reduced in cells expressing the HHH mutant, the joints have wild type levels of nucleotide loss. This reinforces the conclusion that it is the failure of a DNA end to engage the DEB that promotes nucleolytic end-processing. So, even though the HHH mutant impairs the ability of the kinase to become fully activated, this failure is not sufficient to promote nucleolytic end-processing. The few joints amplified from cells expressing the combination mutant are like those amplified from YARK>AAAA expressing cells.

Stable clones expressing these mutants were established; cells expressing the HHH mutant alone or in combination with the YARK>AAAA mutation are as sensitive to calicheamicin as cells lacking DNA-PKcs. DNA-PKcs autophosphorylation was assessed. As noted above, mutation of the DEB helix promotes ABCDE autophosphorylation, but impairs PQR autophosphorylation. The HHH mutation also impairs S2056 autophosphorylation but does not augment T2609 phosphorylation, consistent with not activating excessive nucleolytic activity. In cells with the HHH+YARK mutation, S2056 autophosphorylation is completely blocked whereas T2609 phosphorylation is not reduced.

From these data, we conclude that DNA-PK can be activated in two distinct manners: 1) a partial and transient activation that leads only to ABCDE phosphorylation and rapid kinase inactivation, and 2) full kinase activation that facilitates both ABCDE and PQR autophosphorylation as well as phosphorylation of DNA-PK’s other substrates. DNA-PK is unique among protein kinases in its requirement for open DNA double-stranded ends for its activation; here we demonstrate that three modes of DNA-PKcs interaction with DNA (i.e., between DNA and the HHH, the DEB, and the DNA binding cradle) are required for kinase activation. The DNA binding cradle interactions are required for both types of kinase activation. The reduction in 2056 phosphorylation with both the HHH and DEB mutants suggest that these DNA binding motifs are required for full kinase activation; moreover, the severe celllular phenotypes associated with these mutations are consistent with impaired formation of the DNA end protection complex which in cryo-EM structures is associated with full kinase activation^10,15,16^. Mutation of the DEB revealed that it is the failure of the DEB interaction with the DNA end that results in the partial and transient activation of DNA-PK; studies of DNA-PK activation by hairpin ends predicted this mechanism^10^. DEB mutation results in both hyper activation of ABCDE sites and dysregulation of end processing in living cells. Thus, it is the DEB/DNA interaction (or lack thereof) that differentially activates the kinase resulting in DNA-PK’s capacity to regulate end processing.

### DEB mutants are rescued by promoting formation of the XLF-mediated dimer.

In our recent study of DNA-PK mediated dimers^14^, we showed that two different DNA-PK dimer complexes [XLF-dependet dimer and Ku80-mediated dimer] have distinct functions; morover our studies are consistent with these two dimer forms existing in equilibrium with each other. Thus, although mutations that disrupt either dimer impart distinct cellular deficits in DNA repair, when both dimers are impaired by mutation, cellular NHEJ is substantially restored. It is notable that mutants that disrupt the DEB helix, “phenocopy” mutants that disrupt the XLF-dependent dimer, both showing marked sensitivity to calicheamicin and increased resection of VDJ coding joints. These data suggest that “engagement” of the DEB helix with the DNA end promotes formation of XLF-dependent dimers. We tested this by making mutants that combine the DEB mutations with mutations that block either the XLF-dependent dimer or the Ku80-mediated dimer. The prediction would be that blocking the Ku80-mediated dimer (which promotes formation of the XLF-dependent dimer) would partially rescue the DEB mutation; in contrast, blocking the XLF should not rescue the YARK>AAA mutant.

**Fig. 6.**
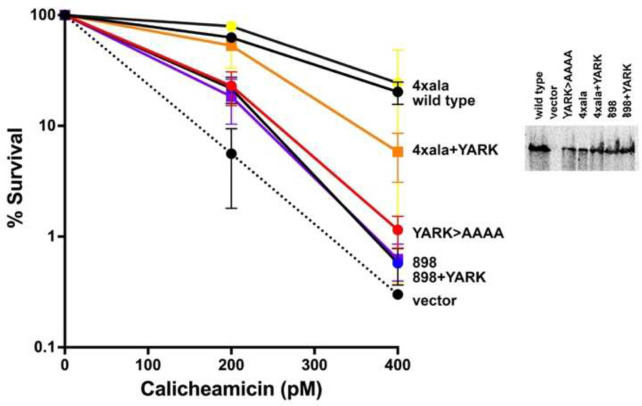
DEB mutants are rescued by promoting formation of the XLF-mediated dimer. (A) V3 clonal survival assays as in Fig. 2. (B) Immunoblot analyses using indicated antibodies of cell extracts from indicated V3 transfectants.

The prediction was correct (Fig. 6); cells expressing the combination YARK>AAAA and XLF-dependent dimer mutation (898) are similarly hypersensitive to calicheamicin as cells expressing the YARK>AAAA mutant alone. In contrast, combining YARK>AAAA with the K80-mediated dimer mutant (4xala) partially restores resistance to calicheamicin. These data support the model that the DEB promotes formation of DNA end protection monomers that progresses to XLF-dependent dimers. To extend these findings, mutants were also constructed combining alanine or aspartic acid substitutions for the PQR autophosphorylation sites. Although it is known that PQR phosphorylation is functionally important, and that PQR phosphorylation limits nucleolytic activity^22^, no mechanistic insight as to how PQR phosphorylations impact repair has been suggested. We considered that PQR phosphorylations might promote stability of the XLF-dependent dimer; this hypothesis is bolstered by a recent report showing a requirement for PQR phosphorylation in XLF deficient mice^29^. Similar to the mutant that disrupts the Ku80-mediated dimer, phospho-mimicking PQR mutants substantially rescue cells from the impact of the YARK>AAAA mutation whereas phospho-blocking mutants potentiate the effect (Sup. Fig. 4). Moreover, this effect is independent of Artemis. These data suggest that PQR phosphorylation facilitates joining by stabilizing the XLF-dependent dimer. All together, these studies suggest that the ability or failure of DNA ends to stably interact with the DEB helix promotes formation of the XLF-dependent dimer or K80-mediated dimer respectively.

## Discussion

Here, using a mutational approach, we interrogate the mechanism by which DNA ends activate the DNA dependent protein kinase. Three distinct DNA/protein interactions are required. 1) Stabilization of the DNA (internal to the DNA end) is required for kinase activation; stabilization is mediated by four patches in the N-HEAT region that generate the DNA binding cradle which is extended by interactions with Ku. 2) Interaction of the DNA terminus with the DEB is central for kinase activation. Opened DNA ends are split around the DEB helix; the interaction of the 3’ end is stabilized by a basic patch (residues 2727–2731); mutation of either the DEB or this basic patch results in indistinguishable cellular phenotypes that include reduced end joining and excessive nucleotide loss from joined ends. 3) The recently described HHH motif interacts with the DNA phosphate backbone of the DNA bound in the DNA binding cradle, stabilizing the interaction of the DNA terminus with the DEB. The HHH motif allosterically communicates the signal to activate via communication on its opposite face with the extreme N-terminus of DNA-PKcs.

**Fig. 7.**
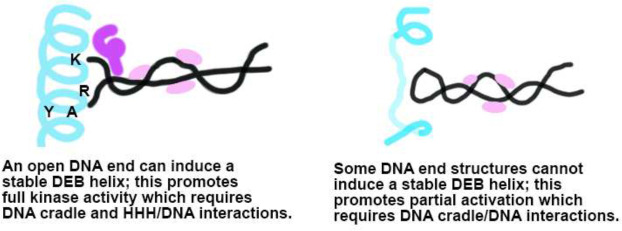
Model of two modes of DNA-PK activation. Cartoon of DEB is colored blue, DNA cradle patches pink, and HHH motiff purple.

Disruption of any of these three protein/DNA interactions impacts kinase activation, but in different ways. If the DNA terminus can split around the DEB, DNA-PK forms an end-protection monomer that is active towards DNA-PK’s many substrates. Full DNA-PK activation requires all three DNA-protein interaction motiffs (Fig. 7 left panel). If the DNA terminus cannot stably interact with the DEB, the kinase is only partially and transiently activated, promoting only ABCDE autophosphorylations that promote nucleolytic end-processing. This activation requires the DNA cradle interactions but niether the HHH or DEB interactions are strictly required for this partial activation. However, a **lack** of interaction of the DNA end with the DEB is specifically required to promote this partial kinase activation that activates nucleolytic end-processing (Fig. 7 right). Finally, since DEB mutation promotes nucleolytic end processing in living cells, it follows that mutations that disrupt DEB function might result in DNA-PK mutants that promote excessive genome mutation because of increased level of nucleolytic activity during repair. Indeed, mutations impacting the DEB helix have been reported in various human cancers (TCGA database).

What is still unclear is what promotes formation of the lone DEB helix in some DNA-PK complexes, but not others. Unpacked alpha helices are extremely rare in protein structures; however, a recent study documents a similar unpacked helix in the N-terminal disordered region of the H1 linker histone^30^. This helix is actually induced by DNA binding, and the authors propose that it is the interaction of basic residues in this DNA-binding induced helix that facilitates its stability. In the many cryo-EM structures that have documented the DEB helix, it is clear that the DNA ends interact with the residues 2743–2746 (YARK) so a similar DNA-basic residue interaction might facilitate stability of the DEB.

The observation that both the ability of the DNA end to be split, or not to be split around the DEB lead to kinase activation is novel and provides a clear explanation as to how DNA-PK regulates DNA end processing. These data clarify previous studies^21,22,31,32^ showing that ABCDE phosphorylation is required to promote nucleolytic end processing [now known to be mediated by the opening of the DNA binding cradle and activation of Artemis^10^] whereas PQR phosphorylation severely limits nucleolytic end processing (shown here to promote formation of the XLF-dependent dimer). Here, we posit that the failure of DNA ends to split around the DEB leads to inefficient progression to the XLF-dependent dimer; formation of this dimer is required for NHEJ^14^. Failure of the DNA ends to split around the DEB leads to transient activation, and promotion of nuclease complexes that we suggest can occur in Ku80-mediated dimers that we have shown to promote nucleolytic activity. The Ku80-mediated dimer likely supports end processing not only by Artemis, but also by enzymes that process trapped top2 products (proposed to be Mre11)^14,33^. Our previous finding that when both dimers are disrupted by mutation, function is restored suggests that the two dimers are in equilibrium.

Here we used this rescue strategy to test whether the DEB helix mutants are impaired in progressing to the XLF-dependent dimer; we show that mutations that disrupt the Ku80-mediated dimer (favoring the XLF-dependent dimer) rescue the DEB mutant. What is still unclear is how DSBs are shuttled between the two dimer forms (for DSBs that require both nucleolytic and fill in end processing) and how the XLF-dependent dimers progress to ligation competent short-range complex. Although elegant studies from He and colleagues have observed complexes that arise from ABCDE phosphorylation within the XLF-dependent dimer^16^, it is unclear whether this intermediate complex progresses to the ligation competent short-range NHEJ complex. Thus, there remains a significant gap in knowledge regarding how NHEJ progresses through these many structurally distinct complexes to promote NHEJ’s iterative manner of repair in living cells.

## METHOD DETAILS

### Survival assays.

Clonogenic survival assays were performed in V3 stable DNA-PKcs transfectants. Briefly, two hundred cells were plated for each transfectant into complete medium containing the indicated dose of the indicated drug in 60 mm diameter tissue culture dishes. After 7 to 10 days, cell colonies were stained with 1% (w/v) crystal violet in ethanol to measure relative survival. A minimum of six independent experiments were performed, and results were averaged.

### Live Cell Imaging.

We used single molecule imaging to determine fraction of wild type HaloTagged DNA-PK and DB4 that were chromatin bound in both unperturbed conditions and after DSBs were induced with zeocin. Live-cell single-molecule experiments have been performed on an Olympus microscope with a TIRF/HiLo illumination system and four laser lines (405nm, 488nm, 561nm, and 647nm). The microscope is equipped with an environmental chamber (cellVivo) to control humidity, temperature, and CO2 level, two iXon Ultra 897 EMCCD cameras (Andor), a 100x TIRF oil-immersion objective (Olympus UApo), and appropriate excitation and emission filters. Approximately 60,00 cells were seeded at grown on 24-well glass bottom dishes and imaged after ~24h. Cells were treated for one hour prior to HaloTag labeling with 100 ug/mL Zeocin or DMSO. Cells were sparsely labeled with JFX650 dye. To remove access of JFX650, cells were washed three times with fresh media and incubated for an additional 10–15 min at 37°C (5% CO2). Cells were imaged continuously with the 647 nm laser at 35% laser power for 3000 frames, with 5.8 ms exposure time, achieving 171 frames per second. All experiments have been done in at least a triplicate with at least 15 cells for each experiment. Single-molecule data was analyzed using SLIMFAST in MatLab 2020b. For tracking, the following settings were used for all the proteins: Exposure Time = 5.8 ms, NA = 1.49, PixelSize = 0.16 μm, Emission Wavelength = 664 nm, Dmax= 5 μm2 s-1, Number of gaps allowed = 2, Localization Error = 10–5, Deflation loops = 0. Next, we used SpotOn tool to determine the bound fractions for tracked particles. The following settings were used: TimeGap = 5.8 ms, dZ = 0.700 μm, GapsAllowed = 2, TimePoints = 8, JumpsToConsider = 4, BinWidth = 0.01 μm, PDFfitting. We applied a 2-state model with the following settings: D_Free2_1State = [0.5 5], D_Bound2_State = [0.0001 0.5] with an assumption that are or chromatin bound or are freely diffusing. To compare bound fractions, we were performed by two-way ANOVA with Tukey’s posthoc test in GraphPad Prism.

### Ku heterodimer purification.

Briefly, Ku70 and Ku80 expression constructs driven by the CMV promoter (His.tag-MBP-Tev-hKu70 = pMM1671) and His.tag-MBP-Tev-hKu80 = pMM1672) were transfected at 1:1 ratio in suspension 293T cells grown in serum free medium using 1:2 ratio of DNA:PEI. Cells were collected by centrifugation at 500 × g after 2 days at 37°C/8% CO_2_ and lysed in 0.8 M NaCl, 20 mM Tris pH 7.5, 1 mM EDTA, 1 mM DTT, 0.2 % Triton X100, 10% glycerol and protease inhibitors. The lysate was treated by sonication to reduce viscosity and clarified by centrifugation at 10,000 × g for 30 min. The extract was incubated for 2 h at 4°C with amylose resin to recover the MBP fusions, washes once with lysis buffer without Triton X100, washes twice with 0.1 M NaCl, 20 mM Tris pH 7.5, 1 mM EDTA, 1 mM DTT, 10% glycerol and protease inhibitors and eluted by adding 40 mM maltose to the low salt wash buffer. The preparation was treated by TEV protease to cleave the MBP moieties and flushed through a Ni-NTA excel column to removed His.tag-TEV, His.tag-MBP and uncleaved fusions. A final step of purifaction was performed by directly loading the Ni-NTA flow-through on a Sepharose Q column equilibrated with 50 mM KCl, 20 mM Tris 7.5, 1 mM EDTA, 1 mM DTT, and 10 % glycerol, and performing a salt gradient from 50 to 500 mM KCl. The peak fractions containing the Ku70:Ku80 heterodimer at 1:1 were pooled dialyzed against 150 mM KCl, 20 mM Tris 7.5, 1 mM EDTA, 1 mM DTT, and 10 % glycerol, aliquoted, flash frozen in liquid nitrogen at stored at −80°C.

### DNA-PKcs purification and *in vitro* kinase assays.

V3 transfectants expressing Flag-Halo-tagged DNA-PKcs were grown in suspension culture. Pellets from ~2.5×10^8^ cells were lysed by freeze/thaw in low salt buffer [10mM HEPES, 25mMKCl, 10mM NaCl, 1mM MgCl2, 1mMEDTA]; after centrifugation at 10,000g, pellets were rinsed in high salt buffer [50mMTris, 5% glycerol, .2mM EDTA, 10mM MgCl2, and 400mM KCl]. Low salt and high salt fractions were mixed adjusting buffer to 400mM KCl. Lysates were absorbed onto 100ul M2-FLAG-Agarose (Sigma) for two hours, then washed three times in high salt buffer. Flag-Halo-DNA-PKcs was eluted overnight by co-incubation with FLAG peptide (200ug/ml) (Sigma). Kinase assays were performed in 50mM HEPES, 100mM KCl, 10mM MgCl2, 0.1mMEDTA, 1mM DTT with 150ug:2, ~700bp indicated restriction fragment for activation and 200uM ATP. Assays were incubated for 30 minutes at 37°C and stopped by addition of 2X SDS-PAGE buffer. Autophosphorylation and phosphorylation of XRCC4 was assessed by immunoblotting.

### Episomal end joining assays.

RAG expression plasmids have been described previously^34,35^. The fluorescent VDJ coding and signal substrates have been described^26,36,37^. Briefly, to assess coding or signal end joining, the coding joint substrate was co-transfected with plasmids encoding RAG1, RAG2, and DNA-PKcs as indicated into V3 cells as indicated. Joining assays were performed on cells plated at 20–40% confluency into 24-well plates in complete medium. Cells were transfected with 0.125 μg substrate, 0.125ug RAG1, RAG2, and .25ug DNA-PKcs as indicated per well using polyethylenimine (PEI, 1 ug/mL, Polysciences) at 2 μL/1 μg DNA. Cells were harvested 72 hours after transfection and analyzed for GFP and RFP expression by flow cytometry. The percentage of recombination was calculated as the percentage of live cells expressing GFP divided by the percentage expressing RFP. Data represents at least three independent experiments, each including triplicate transfections.

The coding joint substrate was modified so that the coding flanks included the A or T sequences depicted in Supplemental Fig. 3; in addition, the 23RSS was inverted to provide for inversional joining which facilitates PCR amplification of coding joints. In some assays, transfected plasmids were isolated by alkaline lysates 72 hours after transfection. Coding joints from each transfection were amplified with the tagged primers for amplicon sequencing. PCR products were analyzed by agarose gel electrophoresis and were isolated and subjected to amplicon sequencing (Genewiz). Indel and microhomology analysis was performed using a python script generously provided by Dale Ramsden and Adam Luthman^38^.

### Quantification and Statistical Analysis.

Student’s t tests were used to compare recombination rates between cell lines. Statistical analysis was performed using Prism 8 (GraphPad). All details of statistical analysis can be found in the legends of figures 1, 4, and 6. Comparisons were made to wild-type, with the absence of displayed P values indicating no significance.

## Supplementary Material

Supplement 1

## Figures and Tables

**Fig. 3.**
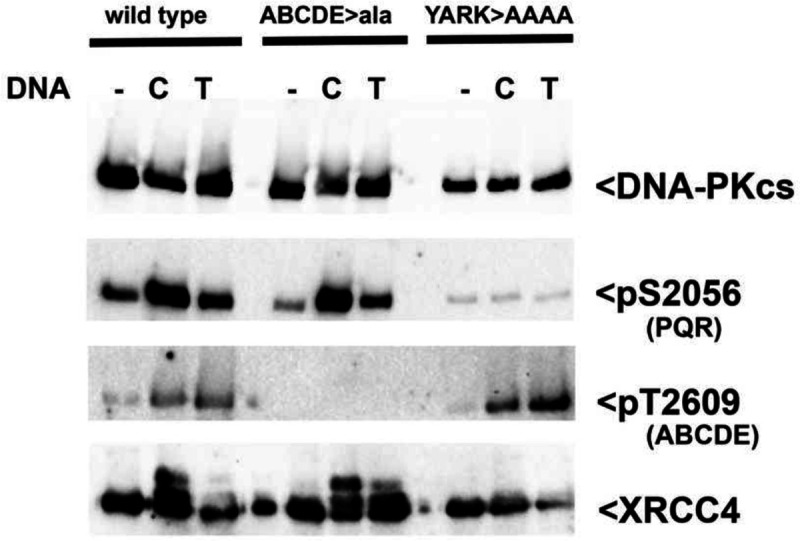
DNA-PK with the DEB helix senses all DNA ends as if they were hairpin ends. (A) Immunoblot analyses of *in vitro* kinase assays.

**Fig. 8.**
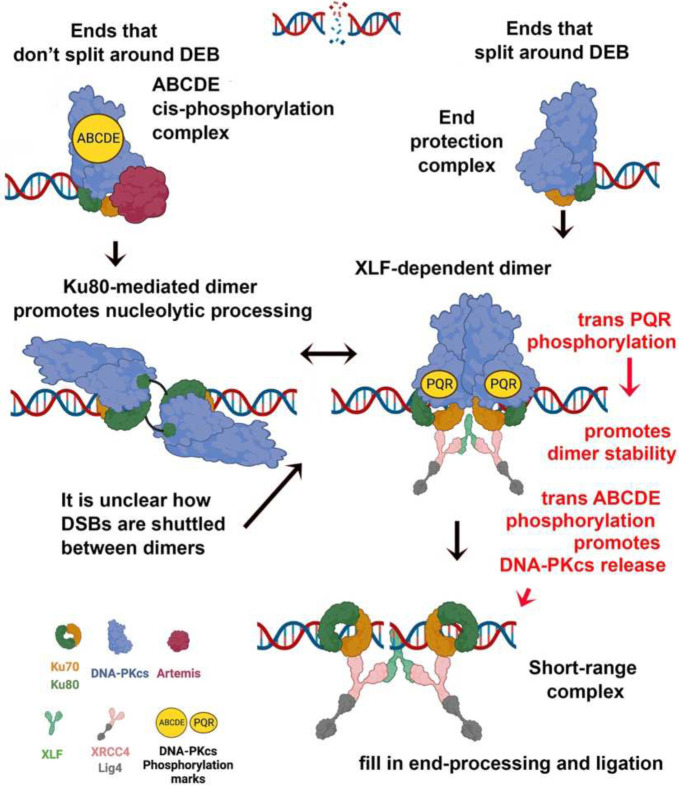
DNA end chemistry dictates which DNA dimer is formed. How DNA ends are shuttled between dimers is not understood.

## Highlights:

- Multiple DNA-protein interactions are required to activate DNA-PK; activation can occur in two distinct ways.
- DNA-PK’s DNA End Blocking (DEB) helix discriminates between DNA ends that require nucleolytic processing versus ends that can be ligated directly or undergo DNA fill-in processing.
- DEB mutation promotes nucleolytic complexes and inhibits formation of the XLF-dependent dimer that is required for NHEJ.
