# Supplementary material for: Unravelling the complexities of DNA-PK activation by structure-based mutagenesis
Source: Res Sq. 2023 Dec 13:rs.3.rs-3627471. Preprint. [Version 1] doi: 10.21203/rs.3.rs-3627471/v1 (PMC10760257; doi:10.21203/rs.3.rs-3627471/v1)
Supplement: Supplement 1 [file NIHPPrs3627471v1-supplement-1.pdf]

Supplemental Information

Supplemental Table 1. DNA-PKcs mutants.

167-169 PDT>AAA	**DB1**	Disrupts DNA-binding cradle 1
215-217 PKL>AAA	**DB2**	Disrupts DNA-binding cradle 2
263-265 KRY>AAA	**DB3**	Disrupts DNA-binding cradle 3
119-122 RAAK>RAPK	**DB4**	Disrupts DNA-binding cradle 4
356-357 NK>AA	**NK>AA**	Disrupts 5' DNA end binding
D405A	**D405A**	Disrupts 5' DNA end binding
518-520 KWK>AAA	**KWK**	Disrupts 3' DNA end binding
2742-2746 MYARK>MAAAA	**YARK or YARK>AAAA**	Disrupts DNA end-blocking helix
2742-2746 MYARK>MDADD	**YARK>DADD**	Disrupts DNA end-blocking helix
2742-2746 MYARK>KKKKK	**MYARK>KKKKK**	Disrupts DNA end-blocking helix
2727-2731 RLRRR>ALAAA	**RLRRR>ALAAA**	Disrupts basic patch across from DEB
2727-2731 RLRRR>DLDDD	**RLRRR>DLDDD**	Disrupts basic patch across from DEB
R820A, K824A, K832A, H833A	**HHH**	Disrupts Helix-hairpin-Helix,
S2023A, S2029A, S2041A, S2053A, S2056A	**PQR>Ala**	blocks phosphorylation of PQR sites
S2023D, S2029D, S2041D, S2053D, S2056D	**PQR>Asp**	phospho-mimics PQR sites
4XK/R>Ala; R1854A +K1857A+ K1913A+ K1917A	**4xala**	Disrupts interaction between DNA-PKcs and Ku80 C-terminus.
2569-2571; S2569A + D2570A	**2569**	Disrupts interaction with DNA-PKcs 898-900
898-900: F898A + R899A + E900A	**898**	Disrupts interaction with DNA-PKcs 2569-2571

Supplemental Figure 1.

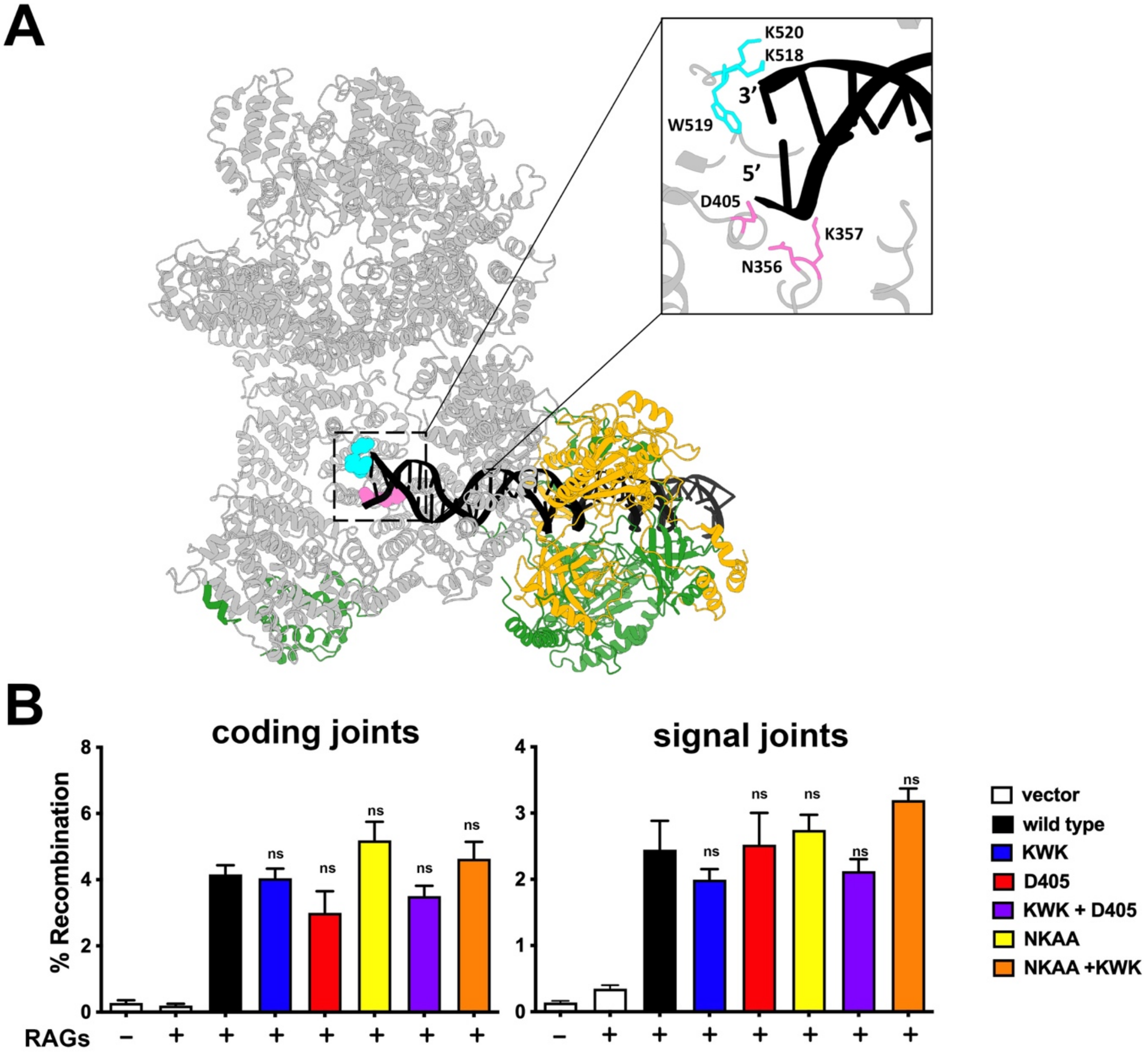

Sup. Fig. 1. Alanine substitution of residues that contact the 5' or 3' DNA end, alone or in combination does not impact joining of VDJ coding or signal ends in episomal assays. (A) Ribbon diagram of DNA-PK monomer (PDB 7K1N). DNA-PKcs is shown in green, Ku80 in orange, Ku70 in gray and DNA in black. Residues contacting the 5' DNA end are shown in red; residues contacting the 3' DNA end are shown in blue. (B) The fluorescent substrate 290-Crimson/ZS and 289-Crimson/ZS were utilized to detect coding and signal end joining of RAG-induced DSBs. Percent recombination of episomal fluorescent coding-end joining substrate in V3 cells transiently transfected with wild-type or mutant DNA-PKcs expression constructs as indicated. Error bars indicate SEM from six independent experiments. t-tests show no signification differences in joining in transfectants with wild type DNA-PKcs compared to each mutant (ns).

Supplemental Figure 2.

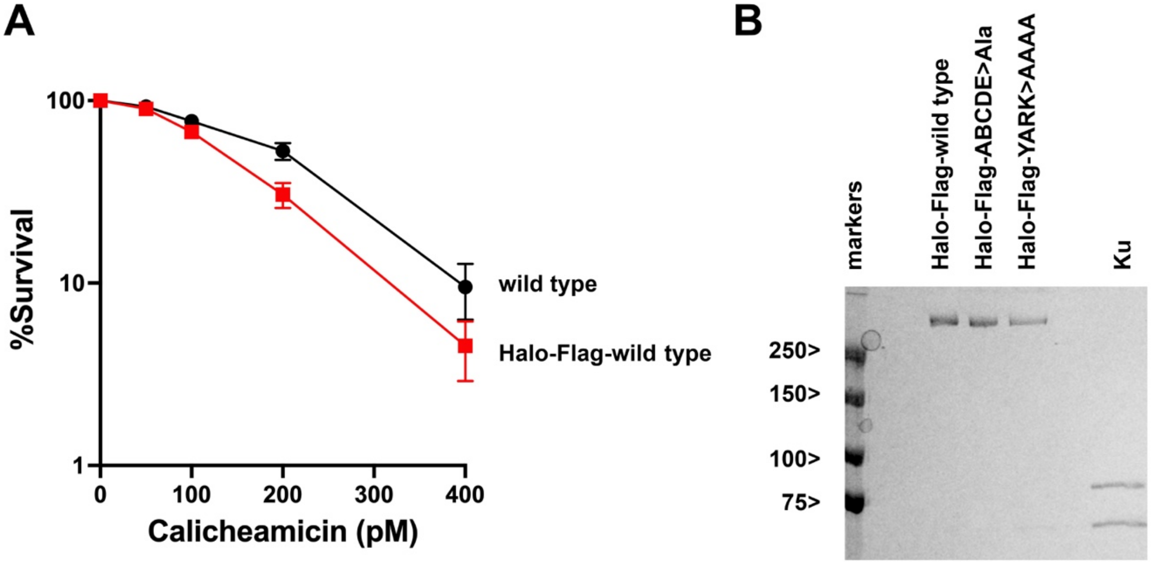

Sup. Fig. 2. Purification of Flag-Halo DNA-PKcs which substantially complements the calicheamicin sensitivity of V3 cells. (A) V3 clonal survival assays as in Fig. 2. (B) Coomassie blue staining of SDS-PAGE analysis of DNA-PKcs and Ku preparations used in kinase assays.

Supplemental Figure 3.

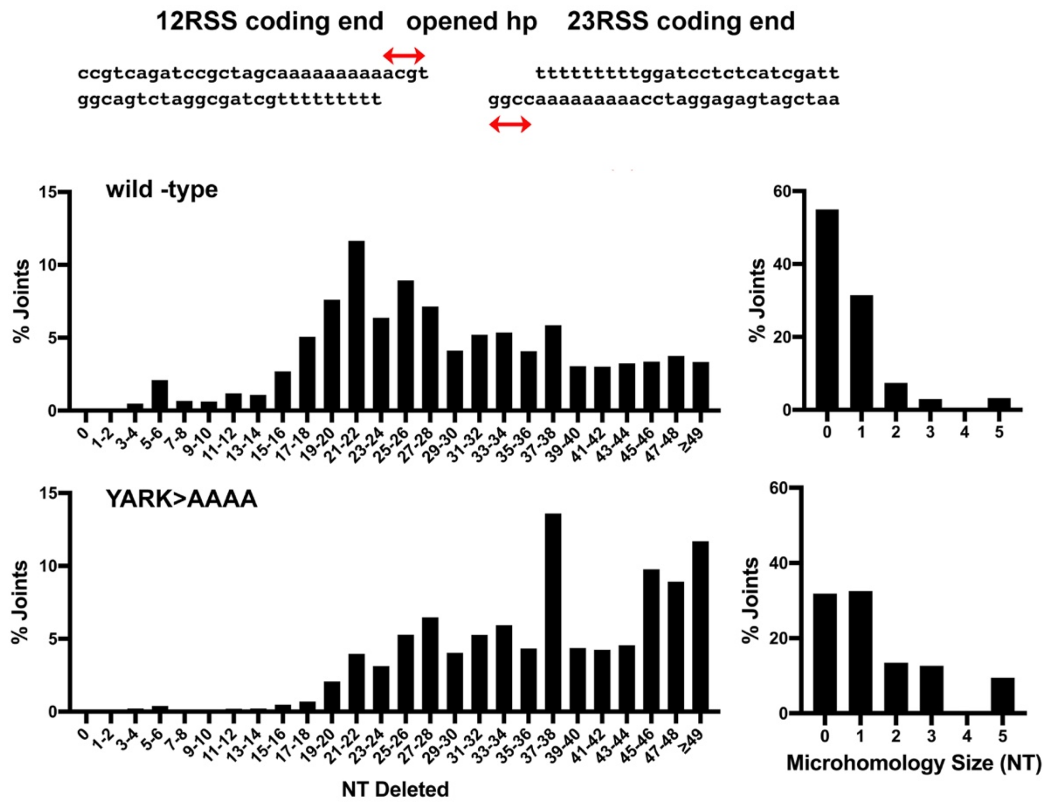

Sup. Fig. 3. Mutation of the DEB helix dysregulates nucleolytic end processing. Summary of amplicon sequencing of coding joints amplified from V3 transfectants expressing wild type or YARK>AAAA DNA-PKcs. Results are averages of two separate experiments.

Supplemental Figure 4.

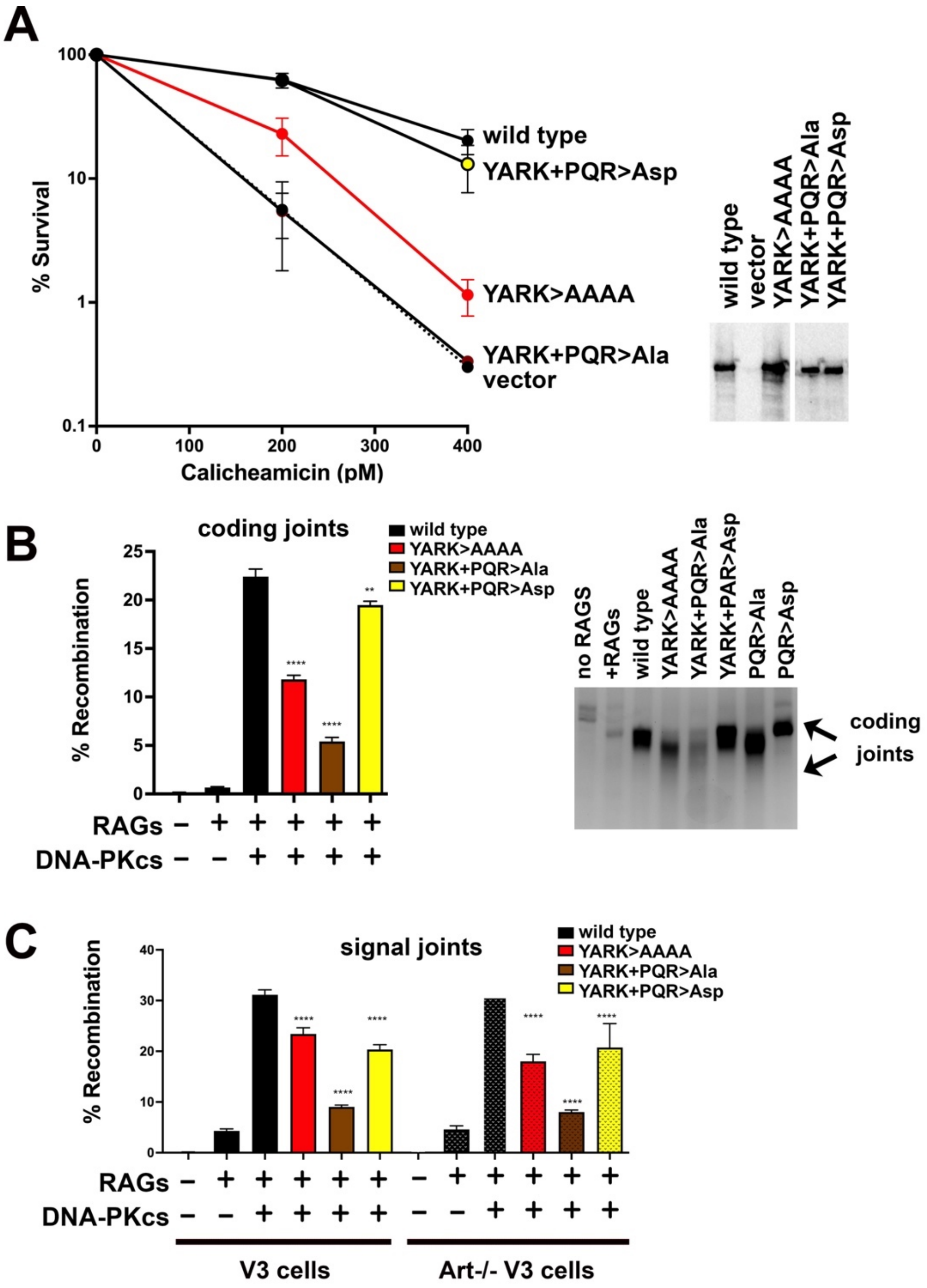

Sup. Fig. 4. PQR phosphorylation rescues the impact of YARK>AAAA mutation. (A) left, V3 clonal survival assays. right, Immunoblot analyses using indicated antibodies. (B) (left) The fluorescent substrate 290-Crimson/ZS was utilized to detect coding end joining of RAG-induced DSBs. (right) 2.5% agarose electrophoresis of PCR amplification of coding joints from AT coding joint substrate. (C) The fluorescent substrate 289-Crimson/ZS was utilized to detect signal end joining of RAG-induced DSBs in V3 cells proficient or deficient in Artemis. t-tests show no signification differences in joining in transfectants with wild type DNA-PKcs compared to each mutant; error bars indicate SEM comparing wild type to each mutant from three independent experiments. ****, P<.0001; ***, P<.001; **, P<.01.
